# Recent advances in conservation and population genomics data analysis

**DOI:** 10.1111/eva.12659

**Published:** 2018-08-20

**Authors:** Sarah Hendricks, Eric C. Anderson, Tiago Antao, Louis Bernatchez, Brenna R. Forester, Brittany Garner, Brian K. Hand, Paul A. Hohenlohe, Martin Kardos, Ben Koop, Arun Sethuraman, Robin S. Waples, Gordon Luikart

**Affiliations:** ^1^ Institute for Bioinformatics and Evolutionary Studies University of Idaho Moscow Idaho; ^2^ Fisheries Ecology Division Southwest Fisheries Science Center National Marine Fisheries Service National Oceanic and Atmospheric Administration Santa Cruz California; ^3^ University of California Santa Cruz California; ^4^ Division of Biological Sciences University of Montana Missoula Montana; ^5^ Département de Biologie Institut de Biologie Intégrative et des Systèmes (IBIS) Université Laval Québec Québec Canada; ^6^ Department of Biology Colorado State University Fort Collins Colorado; ^7^ Flathead Lake Biological Station Montana Conservation Genomics Laboratory Division of Biological Science University of Montana Missoula Montana; ^8^ Wildlife Program Fish and Wildlife Genomics Group College of Forestry and Conservation University of Montana Missoula Montana; ^9^ Department of Biology Centre for Biomedical Research University of Victoria Victoria British Columbia Canada; ^10^ Department of Biological Sciences California State University San Marcos San Marcos California; ^11^ NOAA Fisheries Northwest Fisheries Science Center Seattle Washington

**Keywords:** bioinformatics pipeline, conservation genomics workshop, diversity in STEM, landscape genomics, population genomics

## Abstract

New computational methods and next‐generation sequencing (NGS) approaches have enabled the use of thousands or hundreds of thousands of genetic markers to address previously intractable questions. The methods and massive marker sets present both new data analysis challenges and opportunities to visualize, understand, and apply population and conservation genomic data in novel ways. The large scale and complexity of NGS data also increases the expertise and effort required to thoroughly and thoughtfully analyze and interpret data. To aid in this endeavor, a recent workshop entitled “Population Genomic Data Analysis,” also known as “ConGen 2017,” was held at the University of Montana. The ConGen workshop brought 15 instructors together with knowledge in a wide range of topics including NGS data filtering, genome assembly, genomic monitoring of effective population size, migration modeling, detecting adaptive genomic variation, genomewide association analysis, inbreeding depression, and landscape genomics. Here, we summarize the major themes of the workshop and the important take‐home points that were offered to students throughout. We emphasize increasing participation by women in population and conservation genomics as a vital step for the advancement of science. Some important themes that emerged during the workshop included the need for data visualization and its importance in finding problematic data, the effects of data filtering choices on downstream population genomic analyses, the increasing availability of whole‐genome sequencing, and the new challenges it presents. Our goal here is to help motivate and educate a worldwide audience to improve population genomic data analysis and interpretation, and thereby advance the contribution of genomics to molecular ecology, evolutionary biology, and especially to the conservation of biodiversity.

## 1 INTRODUCTION

At this time, conservation and evolutionary geneticists can employ the power of genomic tools to answer questions in conservation that could not be answered using traditional genetics approaches (Allendorf, Hohenlohe, & Luikart, 2010; Bernatchez et al., 2017; Garner et al., 2016; Harrisson, Pavlova, Telonis‐Scott, & Sunnucks, 2014; McMahon, Teeling, & Höglund, 2014; Shafer et al., 2015a, 2015b). Technological and analytical advances now allow us to use many thousands of loci, gene expression, or epigenetics to address basic questions of relevance for conservation, such as identifying loci associated with local adaptation or adaptive potential in species face changing environments (Bernatchez, 2016; Flanagan, Forester, Latch, Aitken, & Hoban, 2017; Harrisson et al., 2014; Hoban et al., 2016; Hoffmann et al., 2015; Jensen, Foll, & Bernatchez, 2016; Le Luyer et al., 2017; Wade et al., 2016). As conservation genomics matures, new challenges are arising. It is essential for researchers to keep up with the rapidly changing methods in appropriate study design, data quality assessment, and selecting appropriate analyses to obtain accurate results for conservation and management decisions (Benestan et al., 2016).

To address arising challenges, 15 experts from diverse areas of genomic data analysis came together to teach and exchange ideas about cutting‐edge approaches for population genomic data analysis and interpretation. Students, postdocs, faculty, and agency researchers (e.g., museums, agency biologists) originating from 15 countries brought an assortment of data to work through various computational analyses. Of 31 students, 23 had restriction‐site associated DNA (RAD) or genotyping by sequencing (GBS) data, four had exon capture data, and four students had whole‐genome sequencing (WGS) data. Interestingly, of the 30 attendees at ConGen just 4 years ago, only a few students had RAD‐seq data, only one had sequence capture data, and none had WGS data. The main focus of the 15 experts was on narrow‐sense conservation genomics applications, which require use of conceptually novel approaches (Garner et al., 2016).

The week‐long workshop, held at the University of Montana's Flathead Lake Biological Station, provided training in theory as well as empirical applications of NGS data production and analyses. Lectures, discussions, hands‐on analysis of empirical data, and one‐on‐one assistance from instructors improved students’ knowledge of conservation and evolutionary genomic projects. Many participants in the past have taken the knowledge and resources (PowerPoint slides, worksheets, video recorded lectures) acquired during the workshop and disseminated it to others in their laboratories, further extending the educational reach of ConGen among population genomic researchers (http://www.umt.edu/sell/cps/congen2017/).

In the opening keynote lecture, L. Bernatchez discussed several mechanisms that may enhance the maintenance of genetic variation and evolutionary potential in the face of a changing environment. Among these mechanisms that have been overlooked and should be considered in future theoretical development and predictive models, he discussed the prevalence of soft sweeps, the polygenic basis of adaptation, balancing selection, and transient polymorphisms, as well as epigenetic variation. A key message was that adaptive evolution in nature rarely involves the fixation of beneficial alleles. Instead, adaptation apparently proceeds most commonly by soft sweeps entailing shifts in frequencies of alleles being shared between differentially adapted populations. At last, L. Bernatchez argued that a new paradox seems to be emerging from recent studies whereby populations of highly reduced effective population sizes (*N*_e_) and impoverished genetic diversity can sometimes retain their adaptive potential, and that epigenetic variation could account for this apparent contradiction (Bernatchez, 2016).

The remaining lectures focused mainly on approaches for data production or analysis. We discuss highlights from these lectures with the goal of motivating and educating a worldwide audience to improve population genomic data analysis and thereby advance the role of genomics in molecular ecology, evolutionary biology, and conservation. We describe (a) issues regarding recruiting and retaining a diverse workforce in conservation genomics, (b) impacts of genotyping error and data quality, and (c) improvements to downstream population genomic analyses.

## 2 INCREASING CONTRIBUTIONS BY WOMEN (SARAH HENDRICKS AND BRENNA FORESTER)

Following the productive trend at recent ecology and evolutionary biology conferences, issues of gender bias were discussed at ConGen. When this important topic is not widely and openly examined, it can inhibit the advancement of science generally, and conservation and population genomics specifically. Diversity leads to better problem‐solving, expands the talent pool, and promotes full inclusion of excellence across the social spectrum (Blackburn, 2017; Nielsen et al., 2017). Among the plethora of topics regarding increasing diversity in STEM fields (Blackburn, 2017; Wellenreuther & Otto, 2015), here we focus on overcoming the biases against women in computer sciences and the persistence of unconscious gender stereotypes that influence both male and female researchers.

Gender biases in computer science training may limit the effectiveness of efforts to attract and retain the best and most diverse workforce in conservation genomics. As of 2014, just 18.1% of computer science bachelor's degrees were awarded to women, and this proportion has declined by 10% over the last 10 years, further widening the gender gap (NCSES, 2016). This deficit in female computer scientists has been attributed to a lower sense of belonging by women than men due to a predominately male culture in the field (Cheryan, Ziegler, Montoya, & Jiang, 2017). There is also evidence of gender gaps in self‐efficacy that may be due to a lack of sufficient early education in computer programming (Cheryan et al., 2017). Although not reported, these issues likely persist in bioinformatics and genomics. Efforts to maximize gender inclusion in computer science may benefit from changing masculine cultures in technological fields and providing early experiences for all students that signal a sense of belonging and ability to succeed in these fields. Efforts led by women, such as “Girls Who Code” (https://girlswhocode.com/) and “Learn to Code with Me” (https://learntocodewith.me/posts/13-places-women-learn-code/), aim to decrease the gender gap by targeting coding courses and workshops to girls and women. Likewise, short courses such as ConGen, which teach basics in linux, bash, and R scripting, act to support an inclusive community and address limitations due to gendered perceptions in the genomics era.

Unconscious stereotypes persist in the minds of male and female researchers, as evident in the studies of reference letters for postdoctoral fellowships and other academic positions (Dutt, Pfaff, Bernstein, Dillard, & Block, 2016; Madera, Hebl, & Martin, 2009; Trix & Psenka, 2003). One study of recommendation letters for medical faculty positions found that letters written on behalf of females differed from those written on behalf of men in length, negative language, and gender‐linked terms. Overall, the study found that the letters, regardless of the gender of the recommender, reinforced stereotypes that portray men as researchers and professionals and women as teachers and students (Trix & Psenka, 2003). Another study found that men, more than women, were described as having agentic leadership traits, such as being in control of subordinates, speaking assertively, working independently and competitively, and initiating tasks (Madera et al., 2009). Furthermore, women were described as having more communal characteristics, which had a negative association for women with employment decisions (Madera et al., 2009). Letters of recommendation have been shown to greatly affect hireability ratings of applicants (Madera et al., 2009). On the level of personal action, we suggest recommenders edit their own letters to avoid gender bias (http://www.csw.arizona.edu/LORbias).

Despite similar proportions of women and men awarded doctoral degrees in science and engineering disciplines, women are less likely to obtain tenure‐track positions in academia than their male counterparts. Although there are many reasons for this “leaky pipeline” (Gasser & Shaffer, 2014; Goulden, Mason, & Frasch, 2011; Holmes, OConnell, & Dutt, 2015), increasing training and avoiding biases in reference letters may benefit not only women, but also the greater scientific community by promoting innovation through diversity and inclusion. Further, there are many topics such as referee opportunity bias (Lerback & Hanson, 2017), the childcare‐conference conundrum (Calisi & A Working Group of Mothers in Science, 2018), and misconceptions around hiring preferences (Williams & Ceci, 2015) that should also be addressed to reduce disadvantages to women. With the brief mention of this topic, we hope to stimulate future studies of gatekeeping practices in the field of conservation, so institutions can develop initiatives to recruit, retain, and advance women in STEM fields as mentorship will be essential for eliminating gender bias in computer science, bioinformatics, and by extension, conservation biology. We ask our readers to initiate discussions regarding the persistence of stereotypes and how these stereotypes affect excellence across our community. We wonder: Can the active and intentional cultivation of inclusivity help to expand the role of genomics in molecular ecology, population genomics, and nature conservation?

## 3 GENOTYPING ERROR AND IMPROVING DATA QUALITY

On a more technical level, several authors discussed ways to assess and prevent genotyping errors and improve data quality. We discuss several of these here.

### 3.1 Back to the basics: finding and visualizing erroneous data (Eric Anderson and Robin Waples)

#### 3.1.1 Genotyping errors

**Figure 1:**
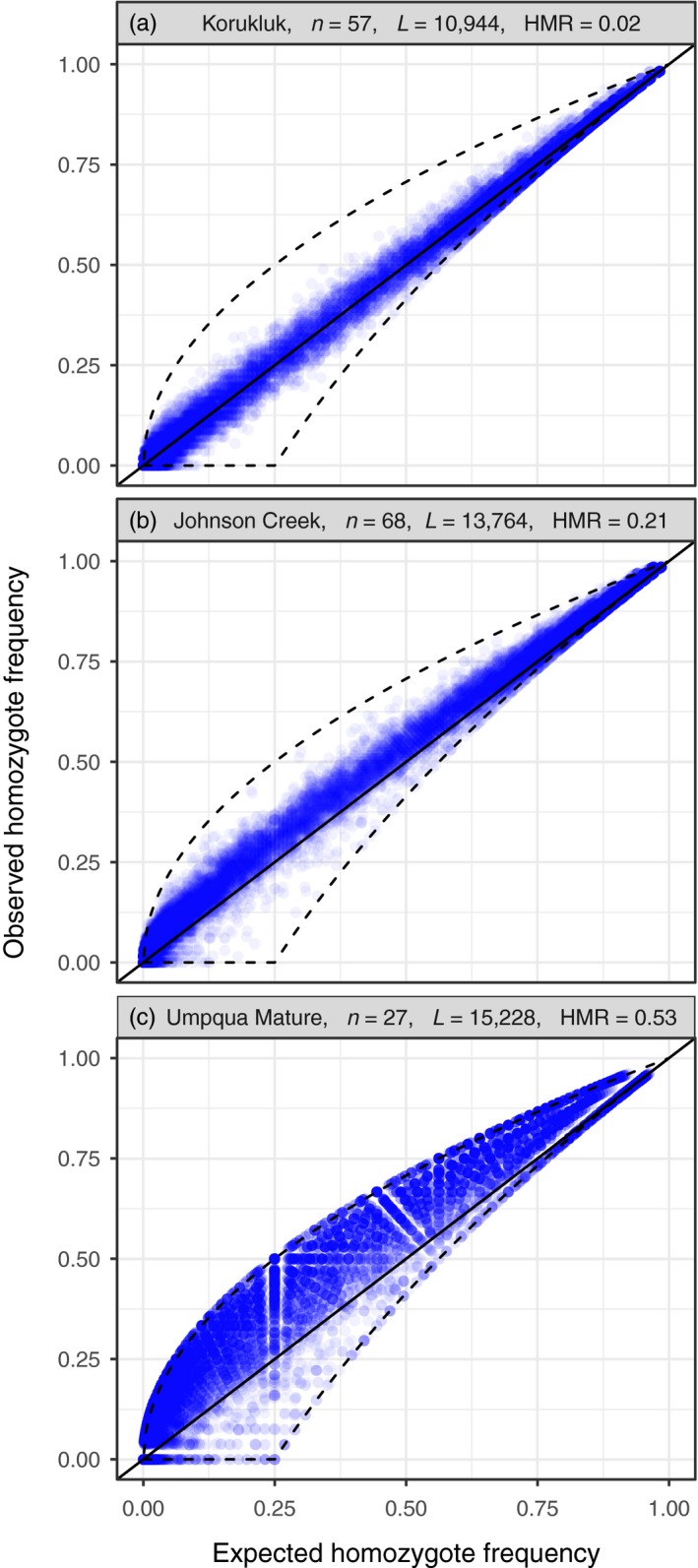
Observed (*y*‐axis) versus expected (*x*‐axis) homozygote frequencies at SNPs in three RAD studies of Chinook salmon. The solid black line is at *y* = *x*, and the dotted lines show the maximum and minimum possible observed values given the expected values computed from the observed allele frequencies. *n* is number of individuals, L is number of SNPs, and HMR is the heterozygote miscall rate estimated from the dataset. (a) Korukluk River, Western Alaska (Larson et al., 2014): a carefully filtered dataset showing almost no distortions from HWE and with a low estimated HMR of 0.02. (b) Johnson Creek (Hecht et al., 2015): Most of the points lie above the *y* = *x* line and HMR is estimated to be 0.17. (c) Low‐read‐depth data from mature‐migrating Umpqua river Chinook (Prince et al., 2017). Genotypes were called using ANGSD's doGeno option assuming a uniform prior on genotypes. Profound homozygote excesses are observed with HMR = 0.52

Systematic departures from Hardy–Weinberg equilibrium (HWE) in datasets where HWE is expected can indicate genotyping errors in which heterozygotes are miscalled as homozygotes. A simple visualization of expected and observed frequencies of homozygote genotypes across single nucleotide polymorphisms (SNPs) can be effective in identifying data problems (Figure 1). A simple model for estimating the heterozygote miscall (dropout) rate was applied to 12 publicly available RAD‐seq datasets (Fernández et al., 2016; Hecht, Matala, Hess, & Narum, 2015; Laporte et al., 2016; Larson et al., 2014; Le Moan, Gagnaire, & Bonhomme, 2016; Portnoy et al., 2015; Prince et al., 2017; Puritz, Gold, & Portnoy, 2016; Ravinet et al., 2016; Swaegers et al., 2015). While a few had low genotyping error rates (<5%), in others, allelic dropout, low read depth, PCR duplicates, erroneous assembly, and/or poor filtering resulted in much higher estimated error rates, with between 5% and 72% of heterozygotes apparently being miscalled as homozygotes. Although some of these apparent high error rates could reflect true heterozygote deficiencies due to the Wahlund effect or other factors, in all cases the samples were thought to be from a single population. Hence, this provides a cautionary note that it is good practice to visualize your data to ascertain if more homozygotes are called than expected under Hardy–Weinberg equilibrium.

#### 3.1.2 Probabilistic genotype calling

Probabilistic genotype calling, as conducted by the software program *ANGSD* (Korneliussen, Albrechtsen, & Nielsen, 2014), is a principled method for dealing with low‐coverage sequencing data; however, it should be applied carefully. With low‐coverage sequencing, because there is so little information at any individual site, the statistical model and the prior distributions are relatively more influential than they are with high‐read‐depth data. A good example can be seen in a recent paper by Prince et al. (2017) which features lower‐depth sampling than many other contemporary RAD‐seq studies. In analyses of their RAD‐seq data, Prince et al. used ANGSD to integrate over the genotype uncertainty rather than directly calling genotypes. Even more importantly, when they were able to, they were careful to use population‐specific allele frequency‐based genotype priors for their analyses rather than a simple uniform prior distribution on genotypes. The choice of prior is important: If one uses ANGSD to call genotypes from the Prince et al. data using the uniform prior on genotypes, the result shows a strong tendency to incorrectly infer heterozygotes as homozygotes (Figure 1c). This is not simply a consequence of forcing ANGSD to call genotypes. Rather, the posterior probabilities, themselves, of the genotypes carry extra weight on the homozygote classes, because the uniform prior does not use allele frequency information to help infer the genotypes.

**Figure 2:**
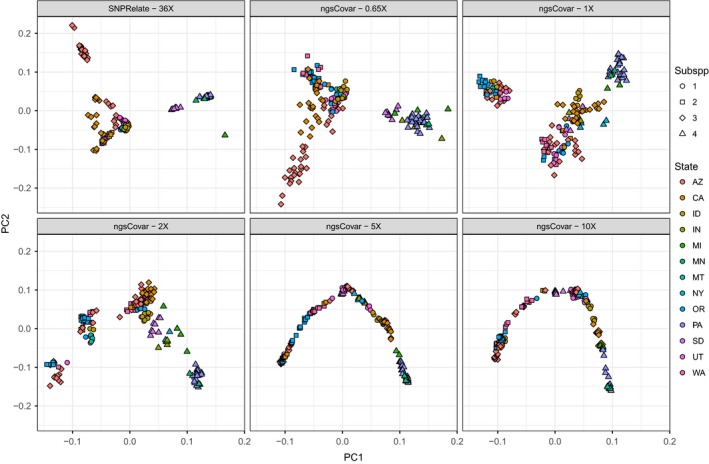
Plots of the first two principal components from PCA of unpublished RAD data showing population structure among four subspecies of a North American passerine. Each point is an individual bird. Top left panel shows the result obtained in the original study, which used 105,000 SNPs called with an average read depth of 36× across 175 birds analyzed with *SNPRelate* (Zheng et al., 2012). Remaining panels show results obtained by subsampling the original dataset to depths of 0.65×, 1×, 2×, 5×, and 10×, and analysis with *ANGSD* (Korneliussen et al., 2014) and *ngsCovar* (Fumagalli et al., 2014). Subspecific structure in the 0.65× data is much less distinct than in the full dataset, but is generally concordant with it. However, at higher read depths the clustering is clearly inconsistent with subspecies affiliation

In an increasing manner, recent publications have suggested that probabilistic genotyping obviates the need for high mean depth of coverage (>10 to 20×). For example, Prince et al. (2017) found that PCA analysis applied to their full dataset yielded a first principal component driven largely by variation in read depth (M. Miller, personal communication, February 7, 2018). Randomly subsampling reads from each individual to the same depth eliminated that technical variation, and, though it led them to discard almost 70% of their sequencing reads, with probabilistic genotyping they were still able to recover meaningful population structure. To evaluate how effectively probabilistic genotype calling can retrieve the same inference with ever‐smaller amounts of sequencing, Anderson presented an analysis using subsampled versions of a high‐depth RAD dataset. He first performed PCA using *SNPRelate* (Zheng et al., 2012) to resolve population structure of a North American songbird using SNPs called from high‐quality, high‐read‐depth RAD data using a GATK pipeline (mean read depth at 105,000 SNPs across 175 individuals was 36). He then used *ANGSD* and *ngsCovar* (Fumagalli, Vieira, Linderoth, & Nielsen, 2014) a probabilistic genotyping approach to PCA, on the BAM files for the same 175 birds after subsampling so that the mean read depth at each of those 105,000 loci was expected to be 0.65, 1, 2, 5, and 10. *ANGSD* was not restricted to using only the previously discovered 105,000 SNPs, and, in fact called between 29,331 SNPs at 0.65× and 898,320 SNPs at 10×. Figure 2 shows that clusters in the first two principal components from *SNPRelate* on the high‐read‐depth data resolve subspecies and show structure within subspecies that corresponds to state of origin. Remarkably, at 0.65×, *ngsCovar* identifies roughly similar groupings, albeit with looser clustering. However, at all other read depths, *ngsCovar* identifies clusters that are clearly inconsistent with subspecies designations and become dominated by Lissajous curves (Novembre & Stephens, 2008).

Overall, the results suggest that some probabilistic methods developed for low‐coverage data might behave unpredictably when provided with high‐quality, high‐read‐depth RAD data. However, new methods based on probabilistic genotyping are continually emerging. For example, the ANGSD methods PCAngsd and PCA_MDS are both reported to outperform ngsCovar with variable sequencing depth (see http://www.popgen.dk/angsd/index.php/PCA). Probabilistic inference from next‐generation sequencing data is an important advance; however, one should not assume that it will automatically overcome shortcomings in sequence data caused by unsatisfactory sample quality, poor library preparation, or insufficient sequencing. As with many approaches for next‐generation sequencing, user‐specified settings of models, priors, and filtering can have strong effects on the results.

#### 3.1.3 Relatedness

Many researchers have concluded that it is important to remove putative siblings from population genetics datasets before conducting downstream analyses (Corlett, 2017; Johnson et al., 2016), but there are several good reasons why this can create more problems than it solves (Waples & Anderson, 2017). First, siblings occur naturally in all natural populations, at frequencies that are inversely related to effective population size; therefore, removing siblings erases signals characteristic of small populations and makes the populations appear to be larger. Second, removing individuals reduces sample size and decreases statistical power, perhaps greatly, so any benefits must be large to offset this cost. Third, methods for sibling inference are not infallible, so it is important to consider the consequences of imperfect pedigree reconstruction. At last, sibling removal cannot be used to convert a nonrandom sample into a random sample, unless one has independent information about the degree to which the proportion of siblings in the sample exceeds the random expectation.

An alternative to removing individuals is to use a best linear unbiased estimator approach (BLUE; McPeek, Wu, & Ober, 2004), which gives each individual a weight that reflects its degree of relatedness to others in the sample. As shown by Waples and Anderson (2017), however, performance of the BLUE also depends on having accurate pedigree information. When sample identification is not reliable, the use of the full dataset outperforms BLUE. Because of these potential adverse effects, researchers should be cautious about adjusting their datasets for putative siblings unless they have a good reason to believe that doing so will not actually make things worse.

### 3.2 Effects of filtering on downstream analyses (Paul Hohenlohe and Tiago Antao)

Methods for producing reduced representation libraries, such as RAD‐seq, are rapidly evolving, and more than 15 methods exist with variations in data quality, genotyping errors, cost, and the number of loci discovered (reviewed in Andrews, Good, Miller, Luikart, & Hohenlohe, 2016). Furthermore, filtering choices (see figure 2 in Benestan et al., 2016) can greatly influence downstream summary statistics. A recent study testing the impact of data processing on population genetic inferences using RAD‐seq data observed large differences between reference‐based and de novo approaches in population genetic summary statistics, particularly those based on the site frequency spectrum (Shafer et al., 2016). In addition, the recent debate over the effectiveness of RAD‐seq for discovering loci under selection (Catchen et al., 2017; Lowry et al., 2016; McKinney, Larson, Seeb, & Seeb, 2017) has highlighted the importance of testing the extent of linkage disequilibrium (LD) over the genome, whenever possible, in order to assess the power of genome scans to detect selected loci (e.g., Kardos, Taylor, Ellegren, Luikart, & Allendorf, 2016).

To further explore the impacts of filtering on downstream analyses, students at ConGen used various minor allele frequencies (MAF; 0.01, 0.05, 0.1, and 0.2) to filter a RAD‐seq dataset and computed *F*_ST_ using the populations function in *Stacks* (Catchen, Hohenlohe, Bassham, Amores, & Cresko, 2013). Participants detected a general trend of increasing estimates of genomewide mean *F*_ST_ with higher MAF thresholds. This may be the result of the relationship between expected heterozygosity and maximum possible *F*_ST_ at SNP loci; given the variation in *F*_ST_ across loci, a subsample of loci with lower MAF may be expected to have a lower maximum and therefore lower mean *F*_ST_ (Roesti, Salzburger, & Berner, 2012). Thus, some filtering by MAF can be used to remove sequencing errors and avoid bias in genome scans (Roesti et al., 2012) and may also remove rare alleles that are less informative for estimating *F*_ST_. On the other hand, imposing MAF filters that are too strict (e.g., above 0.05 or 0.1) could skew metrics based on the site frequency spectrum or inadvertently remove loci under selection or with functional significance. As others have recommended, testing the effects of a range of analytical (filtering) parameters is critical to produce robust population genetic and demographic inferences (Mastretta‐Yanes et al., 2014; Paris, Stevens, & Catchen, 2017; Shafer et al., 2016).

#### 3.2.1 Stringent filtering

The *Anopheles gambiae* 1,000 Genomes Project (Ag1000G) is a large‐scale project to sequence the main vector of malaria, mosquitoes (*Anopheles gambiae*; The Anopheles gambiae Genomes Consortium, 2017), and it has conducted extensive empirical verification of error rates and filtering rules. Parents from different mosquito colonies were mated and produced ~19 offspring for each of four crosses. WGS of all individuals produced a minimum mean coverage of at least 14×. The error rate of SNP variant calling (inferred from parent–offspring inheritance) without filtering was between 13.0% and 21.7%. After filtering, the Mendelian error rate fell to 0.3%–0.9%. The filtering rules devised from this empirical dataset were then applied to the WGS analysis of 765 mosquitoes sampled across Africa. Not using any filtering with GATK would have produced 95,335,499 SNPs, but with optimized filtering rules the number of SNPs fell to 52,525,957 (see Supplementary material of The Anopheles gambiae Genomes Consortium (2017) for filtering parameters). Filtering parameters are dataset dependent and should be modified based on multiple criteria (e.g., depth of coverage, mapping quality, and strand bias) to reduce the number of false discoveries (see GATK forms on applying hard filters for detailed information).

### 3.3 Retaining haplotypes in amplicon and RAD datasets (Eric Anderson)

Common approaches for dealing with multiple SNPs across an amplicon or RAD locus can result in low power or incorrect inference in subsequent analyses. When multiple SNPs are detected, these SNPs are handled as either unlinked (likely untrue) or only one of the SNPs is used in downstream analyses. However, retaining each haplotypic combination as an allele can increase power for relationship inference and pedigree reconstruction (Baetscher, Clemento, Ng, Anderson, & Garza, 2017). Further, haplotype calling allows for the retention of low‐frequency variants, which may be useful for population structure assessment in recently diverged populations. Rare alleles (or haplotypes) reveal recombination events that generated alternative sequences of ancestry and thereby identify fine‐scale structure that would be missed when using independent marker approaches (Lawson, Hellenthal, Myers, & Falush, 2012).

The software microhaplot (https://github.com/ngthomas/microhaplot) takes a variant file and designates nucleotides that occur together on the same read as “microhaplotypes” and allows for the visualization, filtering, and exporting of the data. The *Stacks* software package (Catchen et al., 2013) can also export multi‐SNP haplotypes from RAD‐seq data. Unlike single SNP assays, the microhaplotype data collection method uses assays designed with multi‐allelic loci and can yield useful data for nontarget species phylogenies and for genealogical inference (Sunnucks, 2000).

### 3.4 Draft genomes to improve data analyses (Ben Koop)

Some molecular biologists have claimed that we are in the postgenomic era (Wu, 2001); however, only a very small proportion of reference genomes are assembled to the chromosomal level. Despite this, having even a draft genome (in 1000s of scaffolds) can help improve data analyses in many ways including the following: (a) reliable discovery of SNPs (e.g., avoiding duplicated loci), (b) reducing genotyping error rates (Hand et al., 2015; Shafer et al., 2016), (c) detecting loci under selection by allowing sliding‐window approaches along scaffolds (Hohenlohe, Phillips, & Cresko, 2010), and (d) finding the underlying genes associated with phenotype or adaptation (facilitated by mapping scaffolds to related species with well‐annotated genomes; e.g., Ekblom & Wolf, 2014; Kohn, Murphy, Ostrander, & Wayne, 2006; McKinney et al., 2016). In addition, it is possible with this information to estimate effective population size (*N*_e_; e.g., Li & Durbin, 2011) or effective number of breeders (*N*_b_) using LD‐based methods, as comparisons can be restricted to pairs of loci on different scaffolds, which should reduce or eliminate LD due to physical linkage. Depending on the genome size and complexity, an investment of $10k to $20k could achieve a useful reference genome with an N50 of ~100 kbp, which can be sufficient to improve data analysis as mentioned above (see Goodwin, McPherson, & McCombie, 2016) for costs per Gb for various sequencing platforms). Furthermore, the reference assembly could likely be provided by a commercial company (e.g., DoveTail, https://dovetailgenomics.com/) for this price, as long as the genome is not too large (≫3 GB) or complex (e.g., duplicated, numerous repeats), and if the initial DNA is of high molecular weight (many fragments >15–20 kb).

There are a growing number of approaches for genome assembly using “single molecule real‐time” sequencing (SMRT‐seq) or “synthetic long‐read” sequencing (SLR‐seq) technology (Fuentes‐Pardo & Ruzzante, 2017; Goodwin et al., 2016). The SMRT‐seq technology offered by PacBio (http://www.pacb.com/) produces read lengths of ~10 kbp (some >60 kbp). Oxford Nanopore (https://nanoporetech.com/) and minION also use a single molecule approach to nucleotide identification that passes an ionic charge through a nanoscale hole and measures the changes in current as each molecule passes through (see Michael et al. (2017) for assembly comparison). SLR‐seq technologies, such as 10× Genomics (https://www.10xgenomics.com/) or Dovetail Genomics (https://dovetailgenomics.com/), still rely on short read technology and, using statistical phasing algorithms, have the capacity to assemble continuous haplotypes and scaffolds that can span whole chromosomes with high accuracy.

While the per sample cost of WGS is still relatively high, the per locus cost is low compared to reduced representation library costs (see table 1 in Oyler‐McCance, Oh, Langin, & Aldridge, 2016). A greater proportion of positions within the genome are covered with WGS, which lowers the per base sequencing costs, but increases the costs per individual. With sequencing prices still falling, it is becoming more likely that most ecologists and evolutionary biologists will have access to genome assemblies for their study species (or sister taxa) in the near future (Ellegren, 2014).

### 3.5 Experimental design: which method to choose (Paul Hohenlohe)

The diversity of options for experimental design of population genomic studies continues to expand as sequencing costs continue to drop and new technologies emerge. As discussed in previous ConGen workshops (Benestan et al., 2016), a general guideline is to consider carefully the biological question, and the downstream analyses and statistical power that will be required to most efficiently address it. This should guide all aspects of experimental design, including the genomic approach, type of genetic markers, number of markers, sequencing depth, number of individuals and populations sampled, spatial distribution of individuals, and tissue type (for transcriptome sequencing). For all of these factors, there is a wide range of options for most population genomic studies, as well as trade‐offs among methods and sampling approaches that are important to consider (Andrews et al., 2016; Benestan et al., 2016).

Focusing on the choice of sequencing method, a particular point of discussion at the ConGen 2017 workshop was the recent set of papers addressing the limitations of RADseq to illuminate the genetic basis of adaptation (Catchen et al., 2017; Lowry et al., 2016; McKinney et al., 2017). The primary criticism raised by Lowry et al. (2016) is that RAD loci, depending on the choice of restriction enzyme(s) and the specific protocol used (Andrews et al., 2016), may be sparsely distributed across the genome, so that selected loci may lie some distance away from the nearest genotyped RAD marker. By definition, all reduced representation approaches face this issue, although RADseq approaches are more limited than other techniques (such as sequence capture) in their ability to specifically target previously identified candidate loci. In a RADseq study (and most other marker‐based population genomic studies), the key factor is linkage disequilibrium (LD), which determines the extent to which genotypes at a genetic marker are correlated with those of a functionally important locus, and therefore, the signal of selection that can be detected from marker data.

If the scale of LD is larger than the distance between markers, a RAD‐seq study has a high probability of identifying functionally important loci across the genome. The extent of LD can be directly estimated if a reference genome is available (Catchen et al., 2017), and it is recommended that LD should be estimated whenever possible in population genomic studies. Moreover, many conservation and population genomic questions can be answered without exhaustive sampling of the genome or detection of all functionally important loci, and alternative techniques such as WGS may impose substantial costs and other trade‐offs (Catchen et al., 2017). In particular, increasing the density of markers may necessitate reducing the number of individuals or populations sampled, and choosing methods that target candidate loci can bias against detecting selection at previously unknown loci. Overall, there is no universally applicable genomic method, and the biological question and details of the study system should drive the choice of technique.

## 4 IMPROVING DOWNSTREAM COMPUTATIONAL ANALYSES

### 4.1 Genomic analysis of inbreeding and demographic history (Marty Kardos)

In a traditional manner, individual inbreeding has been measured with the pedigree inbreeding coefficient (*F*_P_) via path analysis (Pemberton, 2008). More recently, large numbers of genetic markers (Berenos, Ellis, Pilkington, & Pemberton, 2016; Hoffman et al., 2014; Huisman, Kruuk, Ellis, Clutton‐Brock, & Pemberton, 2016) and whole‐genome sequences (Kardos et al., 2018; Palkopoulou et al., 2015; Xue et al., 2015) have been used to estimate individual inbreeding directly from the genome by analyzing parameters like multiple‐locus heterozygosity, genomic relatedness matrices, and Runs Of Homozygosity (ROH; Kardos et al., 2016). Genomic approaches capture variation in realized inbreeding that is missed by pedigree analysis due to the stochastic effects of linkage and unknown common ancestors of parents (Franklin, 1977; Thompson, 2013). Thus, while deep and accurate pedigrees can often precisely measure individual inbreeding in species with many chromosomes and/or high recombination rates (Kardos et al., 2018; Knief, Kempenaers, & Forstmeier, 2017; Nietlisbach et al., 2017), genomic approaches are expected to more reliably measure inbreeding and inbreeding depression (Kardos, Luikart, & Allendorf, 2015a; Kardos et al., 2018; Keller, Visscher, & Goddard, 2011; Wang, 2016). Given that many studies have used only shallow pedigrees or few DNA markers, it is possible that power to detect inbreeding depression has been low; therefore, inbreeding depression could be more common, widespread, and severe than previously thought.

Analyses of ROH can also be used to understand the genetic basis of inbreeding depression. Candidate regions for loci contributing to inbreeding depression can be identified as chromosome segments containing fewer ROH in a sample of individuals than expected by chance (Kardos et al., 2018; Pemberton et al., 2012). Homozygosity mapping (Charlier et al., 2008) and association analyses based on the correlation of phenotype with the presence/absence of ROH in particular genome regions (Keller et al., 2012; Pryce, Haile‐Mariam, Goddard, & Hayes, 2014) can be used to identify loci affecting inbreeding depression. Genomic approaches have the potential to greatly advance our understanding of the strength and genetic basis of inbreeding depression in natural populations.

Analyses of identity‐by‐descent (IBD) can also be used to infer historical effective population size (*N*_e_). Differences in historical *N*_e_ among populations can be qualitatively inferred by analyzing the abundance of ROH. The abundance of very short ROH is informative of *N*_e_ in distant history, while long ROH is informative of more recent *N*_e_ (Kardos, Qvarnström, & Ellegren, 2017; Kirin et al., 2010; Pemberton et al., 2012). A limitation of this approach is that it is only qualitative and requires data on multiple populations to be informative.

A particularly exciting new approach for studies of recent demographic history in natural populations is to explicitly estimate a time series of recent *N*_e_ using inference of IBD. The program IBDSeq (Browning & Browning, 2013) searches the genomes of all pairs of individuals to identify chromosome segments of shared ancestry between individuals. The program IBDNe (Browning & Browning, 2015) then uses the inferred pairwise IBD segments to find the most likely recent time series of *N*_e_ given the IBD data. A limitation of this approach for most natural populations is that it requires a minimum of approximately 100 individuals and the genetic mapping locations (i.e., on a linkage map) of at least several hundred thousand SNPs (Browning & Browning, 2015). However, the approach has great potential to infer recent demographic history (i.e., to test for and quantify recent population bottlenecks and expansions) in natural populations where it would be difficult or impossible to evaluate recent *N*_e_ otherwise (Kardos et al., 2017).

### 4.2 Genomewide association studies (Marty Kardos)

Genomewide association studies (GWAS) have recently identified loci with large effects on several ecologically important phenotypic traits. For example, single loci have explained a large fraction of the variance in age of maturation in Atlantic salmon (Barson et al., 2015) and horn development in free‐ranging Soay sheep (Johnston et al., 2011, 2013). In an intelligible manner, some traits are governed largely by variation at individual loci, but these are likely rare among all traits of interest to evolutionary biologists. Many adaptive traits are likely driven by a large number of loci with small effect sizes, low minor allele frequency, and/or epistatic interactions (Visscher et al., 2017). GWAS of complex traits will therefore often fail to identify enough genotype–phenotype associations to explain a useful fraction of the heritability of traits of interest. This is particularly true of studies on populations with very large *N*_e_ or high recombination rates where strong linkage disequilibrium (LD) extends only very short distances from the genotyped loci, or where relatively few loci are analyzed, thus resulting in low power to detect loci even with relatively large phenotypic effects (Kardos et al., 2015b). However, encouraging for studies in small or fragmented populations, the power to detect large effect quantitative trait loci (QTL) is expected to be higher in populations with small *N*_e_ because strong LD extends over longer chromosomal distances in such populations. Therefore, the design and interpretation of GWAS are greatly improved by evaluating the extent of strong LD and the power to detect large effect QTL.

By good fortune, GWAS failing to explain a large fraction of the heritability in loci with statistically significant genotype–phenotype associations are still highly useful. It is arguably more important in ecological and conservation genetics to understand the heritability of a trait than to identify some of the loci responsible for heritable variation in the trait, as it is the heritability of a trait that determines the magnitude of the expected response to selection. The additive genetic variance and heritability can readily be estimated using linear mixed effects models (Rönnegård et al., 2016; Santure et al., 2013; Yang, Lee, Goddard, & Visscher, 2011) in GWAS, even in cases where no individual loci pass the stringent thresholds of statistical significance. In addition, heritability can be partitioned among chromosomes to determine whether the trait of interest is likely to be polygenic (i.e., affected by a very large number of loci), in which case chromosome‐specific heritability is expected to increase with the number of genes on a chromosome (Santure et al., 2013).

Participants at ConGen used the R package, RepeatABEL (Rönnegård et al., 2016), to test for loci associated with clutch size using previously published data from a long‐term study of collared flycatchers (*Ficedula albicollis*; Husby et al., 2015). This helped to familiarize students with data structures, available software, and interpretation of results from GWAS. In addition, analyzing the collared flycatcher data allowed students to consider the importance of accounting for repeated phenotypic measurements when conducting a GWAS. Students were encouraged to critically evaluate effect size estimates from GWAS in light of the Beavis effect (Beavis, 1998), and the “winner's curse” (Kraft, 2008), which state that the effect sizes of loci passing a stringent statistical significance thresholds in QTL mapping or GWAS analyses are often upwardly biased, particularly in studies with low statistical power.

### 4.3 Landscape genomics (Brenna Forester)

Landscape genomics is an emerging analytical framework that investigates how environmental and spatial processes structure the amount and distribution of neutral and adaptive genetic variation among populations (Balkenhol et al., 2017). Landscape genomics is sometimes conflated with genotype–environment association (GEA) analysis, which includes a wide variety of statistical approaches for identifying candidate adaptive loci that covary with environmental predictors (Rellstab, Gugerli, Eckert, Hancock, & Holderegger, 2015). However, landscape genomics includes many other techniques for identifying and analyzing spatially structured, selection‐driven variation, including GWAS across multiple environments, simulation studies, experimental approaches such as environmentally stratified common gardens, epigenetic and transcriptomic studies, and innovative approaches that combine analytical techniques (Berg & Coop, 2014; Lasky, Forester, & Reimherr, 2018; Storfer, Antolin, Manel, Epperson, & Scribner, 2015).

Most importantly, landscape genomics is not just the application of these statistical techniques to identify candidate adaptive variation, but is an approach with a developing theoretical framework linking genomic variation, spatial complexity, environmental heterogeneity, and evolutionary processes (Balkenhol, Cushman, Waits, & Storfer, 2015). The wide range of ecological and evolutionary questions and management issues that can be addressed through this framework was highlighted with recent published examples (Brauer, Hammer, & Beheregaray, 2016; Creech et al., 2017; Lasky et al., 2015; Manthey & Moyle, 2015; Razgour et al., 2017; Swaegers et al., 2015).

With this introduction to landscape genomics, ConGen participants worked on applications of GEA analysis, currently the most widely used landscape genomic technique (Balkenhol et al., 2017). The reasons for the popularity of GEA analyses are practical: They require no phenotypic data or prior genomic resources, do not require experimental approaches (such as reciprocal transplants) to demonstrate local adaptation, and are often more powerful than differentiation‐based outlier detection methods (De Mita et al., 2013; de Villemereuil, Frichot, Bazin, François, & Gaggiotti, 2014; Forester, Lasky, Wagner, & Urban, 2018; Lotterhos & Whitlock, 2015). In particular, participants considered how and why detection rates differed between univariate and multivariate GEAs, exploring the use of latent factor mixed models (Frichot, Schoville, Bouchard, & Francois, 2013) and redundancy analysis (Forester, Jones, Joost, Landguth, & Lasky, 2016; Lasky et al., 2012), respectively. Recent work has shown that RDA is an effective means of detecting adaptive processes that result in weak, multilocus molecular signatures (Forester et al., 2018), providing a powerful tool for investigating the genetic basis of local adaptation and informing management actions to conserve evolutionary potential (Flanagan et al., 2017; Harrisson et al., 2014; Hoffmann et al., 2015). Finally, participants were encouraged to move beyond simply documenting candidate adaptive loci in their datasets, and instead focus on the ecological, evolutionary, and management‐relevant questions that can be addressed by more fully integrating a landscape genomic analytical framework.

### 4.4 Ancestral demography with migration (Arun Sethuraman)

Estimation of ancestral demography, particularly under an Isolation with Migration (IM) model (Nielsen, 2001), is useful for many molecular ecologists and conservation geneticists. A prominent set of tools for this analysis includes IM, IMa, IMa2, and IMa2p (Hey, 2010; Hey, Chung, & Sethuraman, 2015; Hey & Nielsen, 2007; Sethuraman & Hey, 2015). In general, these methods utilize a Bayesian Metropolis‐coupled Markov Chain Monte Carlo (MCMCMC) method to estimate effective population sizes, migration rates, and divergence times under the IM model from haplotypic data. In its latest edition, IMa2p offers parallelized estimation under this framework, providing almost linear improvement in computational time by increasing the number of processors utilized. This in turn allows the analyses of a large number of genomic loci to estimate demographic history, a task that was previously intractable owing to computational overhead. These tools assume that genomic loci are independent, freely recombining between loci, nonrecombining within loci, and putatively neutral (summarized in Strasburg & Rieseberg, 2010). When datasets fit these assumptions, the methods give robust results (summarized in Sousa & Hey, 2013). As of late, Hey et al. (2015) simulated data where the number of loci sampled was small and exhibited very low divergence between populations to detect an excess of false positive for the presence of migration (also described in Cruickshank & Hahn, 2014). This study points to high false‐positive rates for detecting migration using likelihood ratio tests while using the IM suite of tools on data that show low divergence (i.e., very low *F*_ST_), and while using a small number of loci. In addition, much like other MCMC methods (e.g., STRUCTURE; Pritchard, Stephens, & Donnelly, 2000), the length of a “run” of the IM tool used is paramount (longer and many duplicate runs preferred) in ensuring mixing, convergence, and adequate sampling of genealogies.

Recent studies that have estimated demography under the IM model as applied to conservation include McKelvy and Burbrink (2017) that tests 24 nested models of evolution and species delineation across the North American range of the yellow‐bellied kingsnake (*Lampropeltis calligaster*), and Vázquez‐Miranda et al. (2017) study of Le Conte's thrashers (*Toxostoma lecontei*) in estimating negligible migration among subspecies to recommend conservation status across their Western North American range. Other tools to test complex demographic models using genomic data include coalescent simulation‐based methods (e.g., FASTSIMCOAL; Excoffier & Foll, 2011; Excoffier, Dupanloup, Huerta‐Sánchez, Sousa, & Foll, 2013), Approximate Bayesian Computation (ABC; Beaumont, Zhang, & Balding, 2002; Robinson, Bunnefeld, Hearn, Stone, & Hickerson, 2014)‐based methods that compare summary statistic distributions in simulated versus observed populations, and diffusion approximations to the joint allele frequency spectrum for demographic inference (e.g., ∂*a*∂*i*, Gutenkunst, Hernandez, Williamson, & Bustamante, 2009). In general, model‐based estimation of evolutionary demographic history (both ancient and recent) when applied in combination with summary population genetic statistics as described above (including *F*_ST_, inbreeding coefficients, and homozygosity), and non‐model‐based methods (including STRUCTURE and ADMIXTURE; Alexander, Novembre, & Lange, 2009; Pritchard et al., 2000) can prove to be useful means to bridge genomics and conservation in particular.

## 5 BROAD RECOMMENDATIONS AND CONCLUSIONS

Common advice among instructors was to gain extensive experience in computer programming. Students were encouraged to seek out online resources and to work in interdisciplinary teams, where through mentorship and close collaboration they can learn the basics in an applied setting. A key theme was the importance of continuing to develop and teach programming at all levels (e.g., elementary through graduate), with a specific focus on better integrating bioinformatics instruction into undergraduate life sciences education.

The advent of “big data” presents a critical challenge in the fields of population and conservation genomics. Interdisciplinary collaboration is a key as it becomes more difficult for researchers to be experts in both data production (e.g., field work, biological sampling) and bioinformatics or mathematical modeling. Koop acknowledges that he fills his team with bioinformaticians as well as biologists, but “when you find the rare individual who understands both the population genomics and the bioinformatics, you do everything you can to hold onto them.” Furthermore, the “Ten Simple Rules for a Successful Cross Disciplinary Collaboration” by Knapp et al. (2015) is a useful resource for gaining skills for a successful, synergistic collaboration.

In conclusion, the genomic era presents both new data analysis challenges and opportunities to visualize, understand, and apply population genomic data to conservation in novel ways. Here, we emphasize producing and visualizing erroneous datasets, possible effects of filtering on downstream analyses, and how to improve downstream computational analyses to prevent drawing erroneous conclusions. The experts at ConGen instructed students to understand and use reliable biological models and to develop clear questions and hypotheses rooted in evolutionary and ecological theory. In summary, ConGen and this article present problems and solutions with the goal of improving the use of genomics in the fields of population genomics, molecular ecology, and conservation biology.

## CONFLICT OF INTEREST

None declared.
